# Effects of tDCS of the DLPFC on brain networks: A hybrid brain modeling study

**DOI:** 10.1371/journal.pcbi.1013486

**Published:** 2025-09-16

**Authors:** Yanqing Dong, Jing Wei, Songjun Peng, Xinran Wu, Yaru Xu, Jianfeng Feng, Jie Zhang, Viktor Jirsa, Jie Xiang

**Affiliations:** 1 College of Computer Science and Technology (College of Data Science), Taiyuan University of Technology, Taiyuan, Shanxi, People’s Republic of China; 2 School of Information, Shanxi University of Finance and Economics, Taiyuan, Shanxi, People’s Republic of China; 3 Institute of Science and Technology for Brain-Inspired Intelligence, Fudan University, Shanghai, People’s Republic of China; 4 Key Laboratory of Computational Neuroscience and Brain-Inspired Intelligence, Fudan University, Ministry of Education, Shanghai, People’s Republic of China; 5 Institut de Neurosciences des Systèmes (INS), INSERM, Aix-Marseille Université, Marseille, France; Ben-Gurion University of the Negev, ISRAEL

## Abstract

Transcranial direct current stimulation (tDCS) has shown promise in treating neurological disorders, particularly through dorsolateral prefrontal cortex (DLPFC) targeting. However, the effects of DLPFC-tDCS on brain functional networks and the underlying propagation mechanisms remain poorly understood. We present a novel tDCS hybrid brain model (tDCS-HBM) that incorporates tDCS-induced gray matter electric fields into a large-scale brain network model, considering their relationship with membrane potential to effectively predict spatiotemporal dynamics. Using this model, we simulated brain activity in response to tDCS over the left (F3-Fp2) and right DLPFC (F4-Fp1). Our results demonstrate that tDCS enhances brain complexity and flexibility, leading to increased functional connectivity (FC) across the whole brain and an improvement in global network efficiency. Dynamic analysis reveals an initial FC decline, followed by widespread enhancement originating from inferior and orbital frontal regions. Importantly, right DLPFC-tDCS induces strong FC associated with the ventral attention network. These changes in topological metrics and spatiotemporal patterns are consistent with prior modeling and empirical findings, validating the utility of our tDCS-HBM in understanding propagation mechanisms. Our hybrid model holds the potential to predict the stimulation effects of modulation protocols, providing precise guidance for clinical neuromodulation interventions.

## Author summary

Non-invasive brain stimulation techniques are increasingly being explored as additional treatments for neurological and psychiatric disorders. However, due to individual anatomical differences and the incomplete understanding of the underlying mechanisms of brain stimulation, there is still no general agreement on the optimal stimulation protocols. Transcranial Direct Current Stimulation (tDCS), a non-invasive brain stimulation technique, modulates brain activity by applying low-intensity electrical currents through electrodes placed on the scalp. In this study, we proposed a computational model of tDCS-HBM (tDCS-Hybrid Brain Model) to predict brain responses to tDCS and provide theoretical support for the optimization of stimulation targets. Using this model, we systematically evaluated the effects of different tDCS stimulation protocols. The results indicated that tDCS enhanced global brain efficiency and functional connectivity, particularly with stimulation of the right dorsolateral prefrontal cortex (DLPFC), which strengthened the functional connectivity of the ventral attention network with other subnetworks, potentially improving alertness. Furthermore, we found that brain responses to tDCS were largely modulated by structural connectivity, providing new insights into the optimization of tDCS treatment protocols. Our approach offers theoretical guidance for the personalized implementation of clinical non-invasive brain stimulation treatments, contributing to the advancement of precision medicine.

## Introduction

Transcranial direct current stimulation (tDCS) shows great potential as a safe and non-invasive way to modulate brain activity and behavior in basic research and clinical applications [1]. tDCS operates by placing electrodes on the scalp, where a constant weak current is delivered to specific brain regions, inducing a depolarization or hyperpolarization of the resting state membrane potential [2]. Depolarizing the resting membrane potential, which enhances cortical excitability, has been demonstrated to facilitate improvements in cognitive functions [3,4]. Currently, the dorsolateral prefrontal cortex (DLPFC) is commonly used as a target for tDCS modulation to ameliorate neurological disorders such as Alzheimer’s disease [5], Parkinson’s disease [6], and psychiatric disorders like schizophrenia [7], and depression [8] as it is involved in a number of cognitive functions such as attention [9], decision-making [10], working memory [11] and emotion regulation [12].

To date, post-stimulation functional magnetic resonance imaging (fMRI) data from patients were adopted to assess the effects of DLPFC-tDCS [13–15] based on brain network metrics and complexity measures. However, ethical constraints on frequent stimulation and the delayed collection of post-stimulation fMRI data hinder the understanding of the immediate effects of tDCS and its propagation mechanisms [16–18]. Fortunately, computational modeling, as an ethically unconstrained research approach, provides a powerful tool for predicting brain stimulation response activity. Several computational modeling studies have advanced the estimation of cortical electric field (E-field) distributions induced by tDCS protocols. By leveraging tissue-specific electrical properties [19–21], finite element modeling (FEM) precisely quantifies the spatial patterns of E-field penetration into cortical regions. Crucially, these studies demonstrate that FEM-derived cortical E-field distributions can predict tDCS-induced changes observed in fMRI data [19,22,23]. However, static cortical E-field distributions are insufficient to predict the brain’s complex dynamic responses.

To more accurately predict the brain’s complex dynamic activity, several studies have incorporated cortical electric field (E-field) into large-scale brain network models to simulate the intricate spatiotemporal changes in neural activity induced by transcranial current stimulation [24–27]. For example, Merlet’s team combined finite element modeling and neural mass models to construct a simple thalamocortical interaction global model, which reproduces the immediate effects of transcranial alternating current stimulation (tACS) [25]. In contrast to tDCS, tACS delivers alternating currents at specific frequencies to drive and modulate neural oscillations, thereby influencing brain function. However, the lack of structural connectivity (SC) between cortical regions limits the investigation of cortical stimulation effects. Kunze et al. further extended this approach by incorporating SC coupling between cortical regions to construct a large-scale brain network model [26]. They added perturbation voltages, based on the cortical current density distribution calculated by FEM, directly to the average membrane potential of each brain region to simulate the polarization effect induced by tDCS on the resting membrane potential. However, when integrating E-fields into large-scale brain network models, prior studies have not adequately incorporated the experimentally observed linear relationship between electric field strength and physiological membrane potential polarization [28]. This oversight affects the prediction and simulation of the complex dynamic brain activity induced by tDCS.

To investigate the effects of DLPFC-tDCS on brain functional networks and understand its propagation mechanisms, we proposed a tDCS hybrid brain model (tDCS-HBM). First, we employed the FEM for tDCS to precisely compute the spatial distribution of electric field intensity in gray matter regions under F3a-Fp2c and F4a-Fp1c electrode configurations. Subsequently, cortical electric fields were mapped to equivalent membrane potential polarization using the linear relationship between electric field intensity and membrane potential polarization. Finally, by assuming a linear proportionality between membrane potential polarization and baseline activity of synaptic gating variables, we integrated this regulatory term into synaptic gating variable equations within a large-scale brain network model. Through this “electric field-membrane potential-synaptic gating” cascade mapping, tDCS-HBM establishes a complete modeling pathway from physical stimulation parameters to whole-brain dynamic responses, enabling direct simulation of spatiotemporal evolution processes in brain activity induced by specific stimulation protocols. We utilized the Human Connectome Project (HCP) Retest dataset to analyze the effects of left DLPFC-tDCS (F3a-Fp2c) and right DLPFC-tDCS (F4a-Fp1c) protocols on the brain functional network at different levels (global, subnetwork, and nodal). Furthermore, the propagation mechanisms of DLPFC-tDCS were explored by dynamic functional connectivity (dFC) approach [29]. Unlike static functional connectivity, dFC captures temporal variations in connectivity patterns, providing deeper insights into the dynamic reorganization of brain networks following tDCS. Our results showed that the tDCS-HBM provides a valuable tool for understanding the network effects of DLPFC-tDCS and offers scientific insight for evaluating and optimizing the effectiveness of clinical neuromodulation protocols.

## Results

### Simulation results by tDCS hybrid brain model

**Fig 1:**
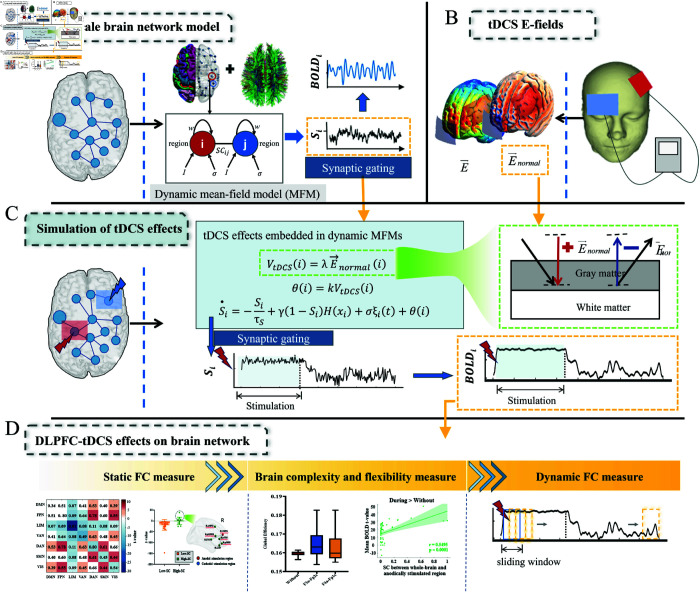
Methodological overview. (A) Building large-scale brain network model. Each parcellated cortical region was modeled using the dynamic mean-field models (MFMs) that express the neural activity of coupled excitatory (E) and inhibitory (I) populations. Interactions between 68 brain regions were coupled by structural connectivity (SC) obtained by diffusion magnetic resonance imaging (dMRI) and tractography. (B) tDCS E-fields. The E-fields were generated using tDCS-FEM and rendered with the SimNIBS software package [30]. The normal component of the E-field is mapped to the gray matter mesh surface. (C) Simulation of tDCS effects. The cortical normal electric field derived from finite element modeling (FEM) calculations was converted into equivalent membrane potential changes via the electric field-membrane potential polarization linear relationship. Subsequently, based on the linear proportionality between membrane potential polarization and synaptic gating variables, the tDCS regulatory factor *θ* was coupled into the synaptic gating variables *S*_*i*_ within the large-scale brain network model. (D) DLPFC-tDCS effects on brain network. The effects of DLPFC-tDCS on brain functional networks were investigated from the perspective of static functional connectivity (FC) metrics (global topological, intra-/inter-resting-state network FC,whole-brain FC) and brain complexity (brain network complexity and flexibility, BOLD-structural connectivity relationship). In addition, the propagation mechanism of tDCS was further investigated using dynamic functional connectivity (dFC).

We proposed the tDCS-HBM to study the brain’s response to tDCS (Fig 1). First, we implemented a large-scale brain network model using SC-coupled dynamic mean-field models (MFMs) to characterize the spatiotemporal dynamic of the brain. Then, we used tDCS-FEM to calculate E-field distributions in the cortex and to identify the crucial brain regions affected by tDCS. Finally, we incorporated the cortical E-fields of the identified crucial brain regions into the large-scale brain network model to investigate the brain response upon stimulation at the network level. To comprehensively analyze the effects of DLPFC-tDCS on brain functional networks, we used the global topological properties, FC within and between resting-state networks (RSNs) and whole-brain FC. To further investigate the effect of stimulation on the brain’s capacity of information processing, we analyzed the complexity and flexibility of brain activity across three periods (Without-tDCS, During-stimulation, and Post-stimulation). We also examined the spatiotemporal dynamics induced by tDCS to investigate how stimulation effects propagate in the brain.

**Fig 2:**
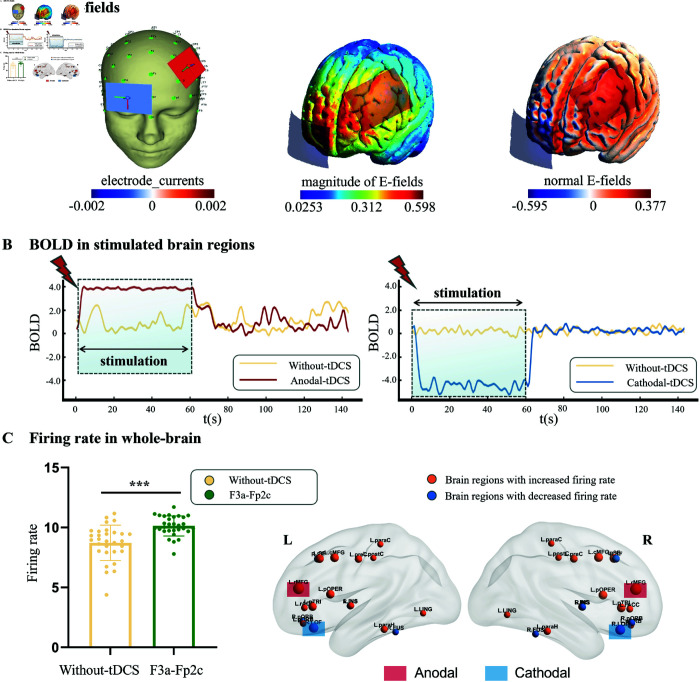
The tDCS-HBM simulation results for the left dorsolateral prefrontal cortex (DLPFC) tDCS protocol (F3a-Fp2c). **(A)** Electric field (E-field) distribution simulated by tDCS finite element model (FEM) and rendered with the SimNIBS software package [30]. **(B)** The changes in the blood oxygenation level-dependent (BOLD) signal in the anodically stimulated region (L.rMFG) and the cathodically stimulated region (R.LOF). **(C)** The average firing rates of Without-tDCS and F3a-Fp2c; brain regions with significant differences are visualized on the right. These results were visualized with the BrainNet Viewer toolbox [31]. *: p≤0.05, **: p≤0.01, ***: p≤0.001.

To understand how the brain responds to stimulation, we conducted a comprehensive analysis focusing on alterations in blood oxygenation level-dependent (BOLD) signal, and firing rates. We found that the BOLD signal in anodically stimulated region (left rostral middle frontal, L.rMFG) increased significantly during stimulation compared to Without-tDCS (Fig 2B). By contrast, an opposite pattern was observed in the cathodically stimulated region (right lateral orbitofrontal, R.LOF), where the BOLD signal were significantly decreased (Fig 2B) compared to Without-tDCS. The change vanished after stimulation stopped, and the BOLD signal recovered to their Without-tDCS levels. The neuronal firing rates (Fig 2C) under the left DLPFC-tDCS (F3a-Fp2c) were higher than Without-tDCS condition.

### The stimulation results for two DLPFC-tDCS protocols

**Fig 3:**
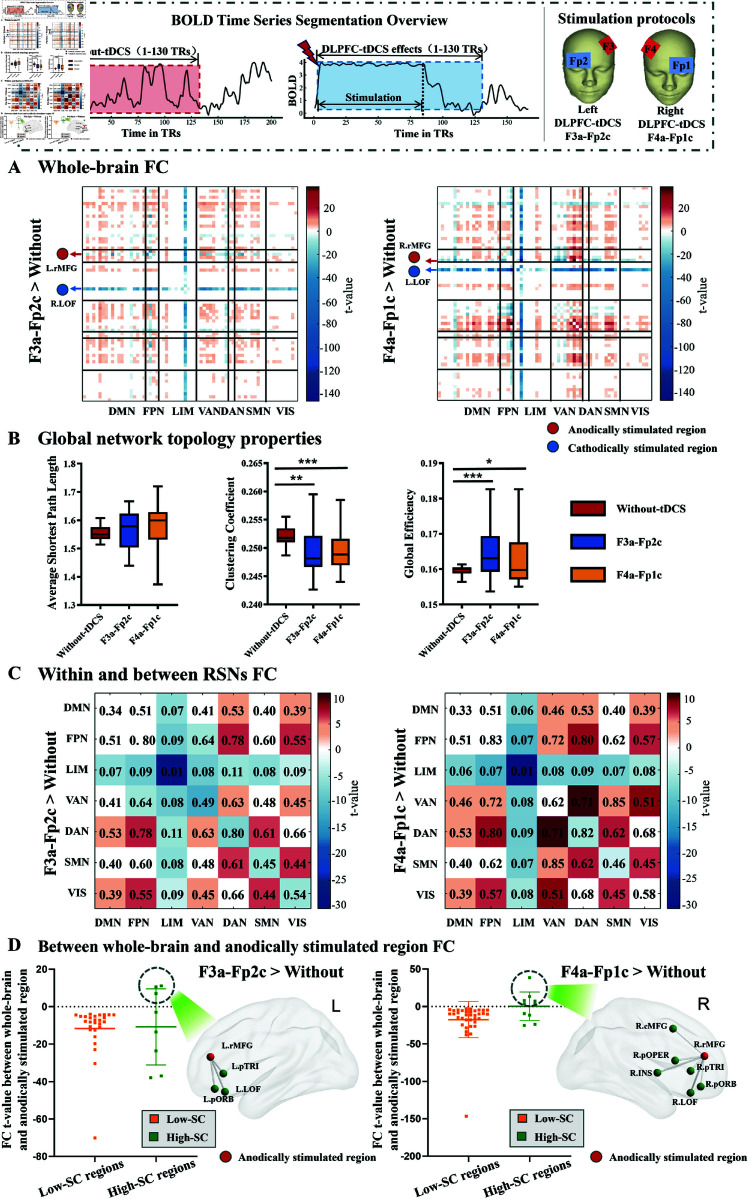
The stimulation effects of two DLPFC-tDCS protocols on brain functional network. **(A)** Changes in functional connectivity (FC) following DLPFC-tDCS compared to Without-tDCS. These results were visualized using the GRETNA toolbox [32]. **(B)** Global topology properties changes for Without-tDCS, left DLPFC-tDCS, and right DLPFC-tDCS. Statistical comparisons were conducted between the two DLPFC-tDCS protocols and Without-tDCS using a paired sample t-test. Data are expressed as Mean ± SD. *: p≤0.05, **: p≤0.01, ***: p≤0.001. **(C)** Changes in FC within and between resting-state networks (RSNs) of two DLPFC-tDCS protocols. Whole-brain FC and FC within and between RSNs were statistically compared between two DLPFC-tDCS protocols and a Without-tDCS condition using a paired sample t-test (p≤0.05). Only nodes with significant differences (paired sample t-test) are colored, while the remaining are white. The color bar indicates the statistical t-value. **(D)** Between whole-brain and anodically stimulated region FC. Brain regions were categorized into “High-SC” (top 10 regions with the stronger SC to anodically stimulated region) and “Low-SC” (remaining 57 regions). Data represent the t-value for significant changes in FC between whole brain and anodically stimulated regions under two DLPFC-tDCS protocols compared to the Without-tDCS condition. These results were visualized with the BrainNet Viewer toolbox [31].

To investigate the induced effects of the left and the right DLPFC-tDCS stimulation on the functional network, we conducted a comprehensive study encompassing global, subnetwork, and nodal analyses. We found that FC exhibited significant increase in most brain regions following stimulation for two DLPFC-tDCS protocols (Fig 3A). There was no significant difference in the average shortest path length for different DLPFC-tDCS protocols compared to the case Without-tDCS (Fig 3B). However, the clustering coefficients decreased after DLPFC-tDCS (Fig 3B) and the global efficiency increased (Fig 3B). The two DLPFC-tDCS protocols (F3a-Fp2c and F4a-Fp1c) showed more consistent results at the global level.

We further investigated the effects of the DLPFC-tDCS at subnetwork levels (Fig 3C). It is evident that the strength of FC within and between limbic network (LIM) have decreased in both two DLPFC-tDCS protocols (Fig 3C). Results obtained with the Destrieux148 atlas showed a similar pattern (see S1 and S2 Figs). The dorsal attention network (DAN) and visual network (VIS) exhibit stronger connectivity with other networks in two DLPFC-tDCS protocols (see Fig 3C). In particular, the right DLPFC-tDCS protocol (F4a-Fp1c) lead to a notable increase in FC between the ventral attention network (VAN) and other networks.

The effect of DLPFC-tDCS was finally evaluated at the node level (Fig 3D). We found that brain regions with strong structural connectivity (SC) to anodically stimulated region showed increased FC after stimulation. In the left DLPFC-tDCS protocol (F3a-Fp2c), the left frontal brain regions, including L.rMFG, left pars triangularis (L.pTRI), and L.LOF, exhibited enhanced FC. In the right DLPFC-tDCS protocol (F4a-Fp1c), there was a greater in FC in the right hemisphere, specifically right caudal middle frontal (R.cMFG), right pars opercularis (R.pOPER), right Insula (R.INS), R.LOF, right pars orbitalis (R.pORB), and R.pTRI.

### Difference in BOLD signal, flexibility, and complexity among three periods

**Fig 4:**
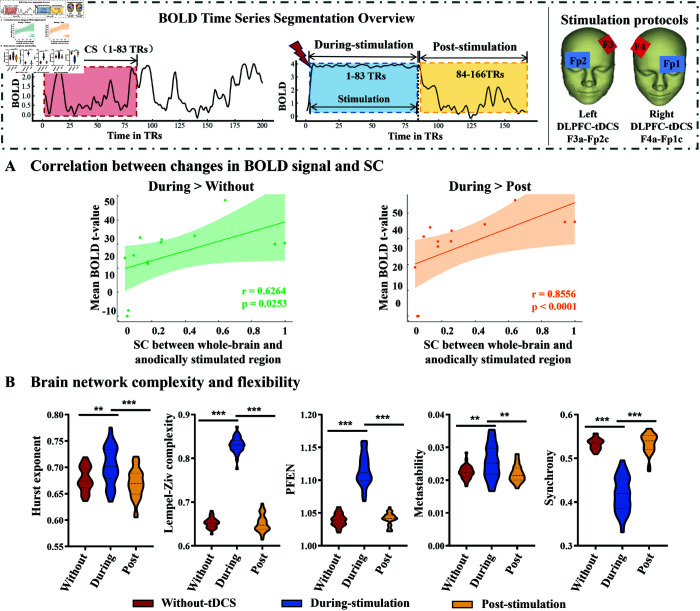
Difference in BOLD signal, flexibility, and complexity among three periods in left DLPFC-tDCS protocol (F3a-Fp2c). **(A)** The correlation between changes in BOLD signal in whole-brain (excluding the anodically and cathodically stimulated regions) across three periods and the strength of their SC to the anodically stimulated region. Vertical axis: t-values from paired t-tests comparing BOLD signal differences between the stimulation period (During) versus baseline (Without) and recovery (Post) periods (During > Without and During > Post contrasts). Horizontal axis: the strength of SC between whole-brain and the anodically stimulated region. **(B)** The complexity and flexibility of the brain across three periods. Statistical comparisons of the Without-tDCS, During-stimulation, and Post-stimulation conditions were performed using paired sample t-test. Data are expressed as Mean ± SD. *: p≤0.05, **: p≤0.01, ***: p≤0.001.

To explore how SC affects the propagation of the observed stimulation effects, we examined the relationship between responses in BOLD signal in each brain region (across different periods) and its SC to anodically stimulated regions (Fig 4A). The results showed a positive correlation between the enhancement of BOLD signal in whole-brain during stimulation (During vs. Without, During vs. Post) and their SC to the anodically stimulated region. However, the t-value of the BOLD change in whole-brain in the Without vs. Post scenario did not correlate with their SC to the anodically stimulated region. This implies that stimulation is delivered along the stronger SC during stimulation.

To further explore the effects of stimulation on the brain’s information processing capacity, we evaluated the complexity and flexibility of the brain during stimulation from various perspectives, including BOLD complexity and entropy, metastability and synchrony (Fig 4B). We discovered significant differences between During-stimulation and Without-tDCS/Post-stimulation. The Hurst exponent, Lempel-Ziv complexity, PFEN, and metastability values were significantly higher in During-stimulation compared to Without-tDCS and Post-stimulation, with synchrony values being significantly lower. These results indicate that the brain state during stimulation is characterized by high complexity and flexibility while low synchrony. The right DLPFC-tDCS protocol (F4a-Fp1c, S3 Fig) also show high complexity and flexibility during stimulation, consistent with the left DLPFC-tDCS protocol (F3a-Fp2c). These results were further confirmed using the Destrieux atlas, which yielded highly consistent findings with those from the analyses based on the Desikan-Killiany atlas (see S4 Fig).

### Propagation of DLPFC-tDCS effects

**Fig 5:**
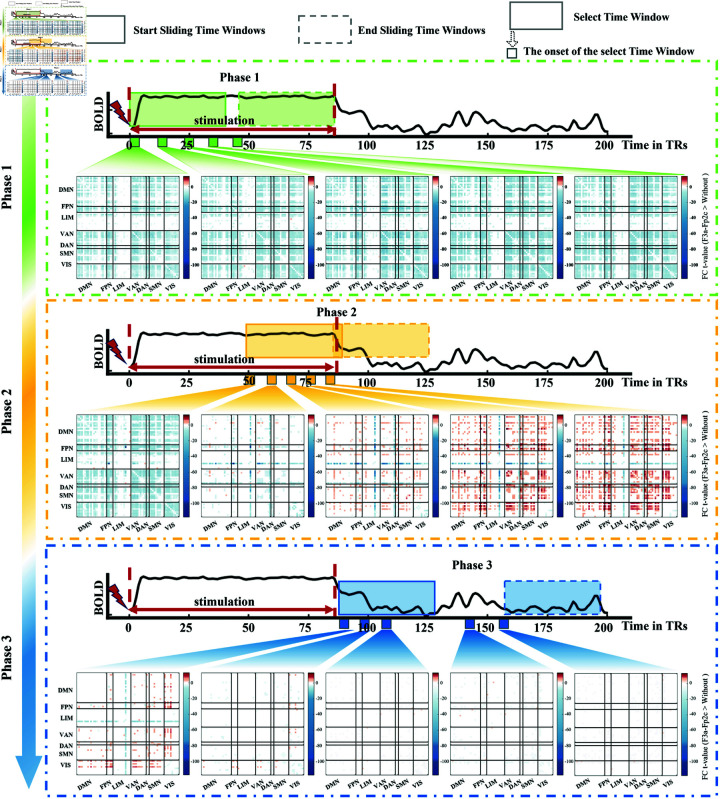
Dynamic functional connectivity changes in left DLPFC-tDCS protocol (F3a-Fp2c). The sliding windows are divided into three phases. **Phase 1:** The window slides throughout the During-stimulation period. **Phase 2:** The window covers the transition from the During-stimulation to the Post-stimulation period. **Phase 3:** The window slides throughout the Post-stimulation period. In each phase, five time-varying FC matrices (depicted as small rectangles in the figure) are shown, where each FC matrix represents the statistical comparison using paired sample t-tests between the left DLPFC-tDCS protocol and the Without-tDCS condition. Only the FC in the left DLPFC-tDCS protocol that is significantly different from Without-tDCS (paired sample t-test) is colored, while the remaining are white. Warmer colors indicate an increase in FC, while cooler colors indicate a decrease in FC. Color bars indicate statistical t-values (p≤0.05).

To elucidate the propagation mechanism of tDCS effects over time, we used the dFC approach to investigate the dynamic FC changes after receiving tDCS (Fig 5). To gain a deeper understanding of the transition process of spatiotemporal dynamics of the brain from During-stimulation to Post-stimulation period, we divided the sliding window into three phases. Phase 1: the window slides throughout the During-stimulation period. Phase 2: the window covers the transition from the During-stimulation to the Post-stimulation period. Phase 3: the window slides throughout the Post-stimulation period. During Phase 1, we observed a whole-brain FC decrease. During Phase 2, the decrease in FC disappeared, followed by an FC enhancement initially in L.pORB and L.pTRI, and finally spread throughout the brain. During Phase 3, the trend of increased FC gradually disappeared and whole-brain FC recovered to resting-state levels. The overall trend of FC decrease and then increase is consistent with the synchrony measure (S5 Fig). The right DLPFC-tDCS protocol (F4a-Fp1c) exhibited a similar pattern of FC change as the left DLPFC-tDCS protocol (F3a-Fp2c), with an initial decrease followed by an increase (S6 Fig). Notably, the analyses based on the Destrieux atlas yielded results highly consistent with those obtained using the Desikan-Killiany atlas (see S7 Fig). Both parcellations exhibited a similar dynamic pattern of functional connectivity changes characterized by an initial decrease followed by a subsequent increase, further supporting the reproducibility of our findings.

### Impact of structural connectivity on dynamic functional connectivity of stimulated regions

**Fig 6:**
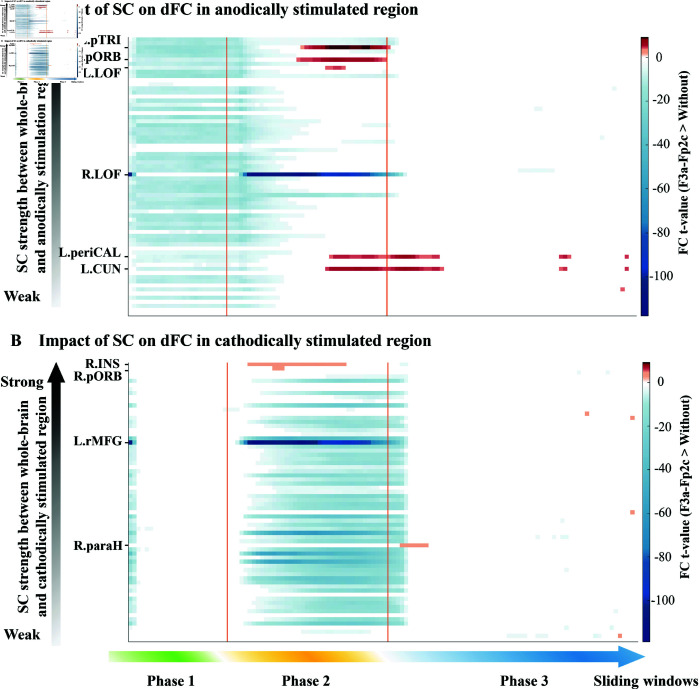
Impact of structural connectivity on dynamic functional connectivity associated with the stimulated regions in left DLPFC-tDCS protocol (F3a-Fp2c). **(A)** Impact of SC on dFC of anodically stimulated region (L.rMFG). **(B)** Impact of SC on dFC of cathodically stimulated region (L.LOF). The horizontal coordinates represent the changes in FC between whole-brain and stimulated brain regions within each sliding window of left DLPFC-tDCS compared to Without-stimulation. The vertical coordinates represent brain regions after sorting from weak to strong (bottom to top) SC strength between whole-brain and anodically or cathodically stimulated regions. Only the FC in the left DLPFC-tDCS that is significantly different from Without-tDCS (paired sample t-test) is colored, while the remaining nodes are white. Warmer colors correspond to an increase in FC, while cooler colors correspond to a decrease in FC. The color bars indicate statistical t-values (p≤0.01).

To further elucidate the role of SC in the propagation of tDCS effects, we examined how the strength of SC between the anodically stimulated region and other brain regions influences the corresponding FC changes (Fig 6). During Phase 2, the FC of two frontal regions (L.pORB and L.pTRI) with strong SC (the strength of their SC to the anodically stimulated region was in the top 10% among all regions) to the anodically stimulated region first increased. Notably, these two brain regions were also the first to exhibit enhanced FC in Fig 5. In addition, we also observed a subsequent enhancement of FC between regions in the occipital lobe (cuneus and pericalcarine) and the anodically stimulated region in both DLPFC-tDCS protocols (Figs 6 and S8).

## Discussion

In this study, we proposed a tDCS-HBM systematically integrates electric field biophysical effects with whole-brain network dynamics through computational modeling. First, cortical electric field distributions derived from FEM were translated into equivalent membrane potential based on an electric field-membrane potential polarization relationship established through in vitro experiments. Subsequently, by introducing a tDCS-specific regulatory term into the governing equations of synaptic gating variables, the model achieves a complete pathway from physical stimulation parameters to dynamic responses across the whole-brain network. We evaluated the effects of tDCS targeting the DLPFC on the brain’s functional network and further investigated the propagation mechanisms of tDCS effects. Both DLPFC-tDCS protocols significantly enhanced global efficiency and subnetwork FC, with right DLPFC-tDCS particularly strengthening the FC between the VAN and other networks. Dynamic analyses further demonstrate an initial decrease in whole-brain FC followed by a gradual increase mediated by SC, which elucidates the propagation mechanism of tDCS. As a computational modeling approach, tDCS-HBM can predict the stimulation effects of different tDCS protocols without ethical constraints, provide details of brain functional networks not captured in clinical experiments, and offer valuable references for predicting the stimulation effects of clinical neuromodulation protocols.

The construction of our hybrid model integrates two key scientific foundations. First, large-scale brain network models accurately capture whole-brain activity dynamics. Second, biological experiments empirically establish a linear relationship between direct current fields and neuronal membrane potential polarization. Specifically, the large-scale brain network model we adopted in this work (68 brain regions coupled through SC, with each brain region described by a dynamic mean-field model) could precisely model observed brain activity (confirmed by our previous study [33]). This framework provides a robust basis for predicting tDCS-induced brain state transitions. Meanwhile, the E-field induced linear polarization of membrane potentials has been validated in an in vivo study, where direct current electric fields were used to modulate neuronal excitability in rat hippocampal slices [28]. Building upon this biophysical mechanism, cortical electric fields were mapped to population-level equivalent membrane potential polarization states, modulating synaptic gating variables to computationally predict tDCS-induced network-level effects. This biological mechanism provides a crucial electrophysiological basis for constructing our model, enabling it to more precisely capture the impact of current stimulation on brain region activity.

The tDCS-HBM replicated the BOLD signal response under different electrode polarities and the increased neuronal firing rates after tDCS, which were consistent with physiological experiments, thereby providing evidence for the validity of the model in simulating physiological indicators under stimulation. Specifically, cathodal stimulation elicits a marked reduction in BOLD-signal amplitude, in agreement with prior experimental observations that cathodal DC stimulation attenuates BOLD-signal amplitude [34,35]. For the increase in neuronal firing rates following stimulation, it was biologically validated in experiments involving direct current stimulation of rat motor cortex slices [36].

The changes in brain network at different scales under both left and right DLPFC-tDCS protocols in our model (Fig 3) were also consistent with a series of clinical trials and empirical studies, which upheld the validity of the model and highlighted the potential of DLPFC-tDCS for cognitive enhancement. At the global level, both DLPFC-tDCS protocols significantly enhanced brain global network efficiency (Fig 3B), which is consistent with the results of clinical trials applying prefrontal tDCS to patients with alcohol use disorders, both indicating the positive impact of prefrontal stimulation on brain function optimization [37]. At the subnetwork level, we observed significant enhancement of FC between the VIS network and other networks (Fig 3C). Lafontaine et al. demonstrated in a tDCS-electroencephalography (EEG) study targeting the DLPFC that prefrontal tDCS facilitates long-range coordination between prefrontal cortices and lower-order visual regions [38]. This facilitation of interregional coordination may mechanistically explain the increased VIS-related functional connectivity observed across networks. The enhanced FC of DAN with other networks in our model was also observed in a study of the modulatory effects of repetitive tDCS on dorsal attention and frontal parietal networks [39]. Finally, we found several regions of DMN and FPN, including the inferior parietal lobule (IPL), precuneus (PCUN), superior frontal gyrus (SF), pars orbitalis (pORB), and rostral/caudal middle frontal gyrus (rMFG/cMFG), all exhibited significantly enhanced FC. This is consistent with previous empirical studies have emphasized the importance of the DMN and the FPN in cognitive function improvement [13,40–44]. This connectivity increase has been proposed to reflect augmented resources and higher readiness to facilitate cognition [45]. Furthermore, we also observed that brain regions with high structural connectivity to the anodically stimulated region exhibited increased FC following stimulation in both left and right DLPFC-tDCS protocols (Fig 3D). The key role of high structural connectivity in tDCS-induced changes in brain activity was confirmed by a computational simulation study, which demonstrated that stimulating the IPL drives brain network activity to a target state, due partially to its high structural connectivity [46]. However, Kurtin et al. pointed out that the brain state has a greater and more generalizable influence on tDCS-induced changes in brain network activity [47]. Vergallito et al. systematically combined transcranial magnetic stimulation with electroencephalography (TMS-EEG), providing evidence of the state-dependent nature of c-tDCS in effectively modulating cortical excitability [48]. These findings collectively suggest that structural connectivity is not the sole determinant of tDCS effects. We speculate that certain brain regions with strong structural connectivity do not show an increase in FC following stimulation, possibly due to the influence of other factors such as brain states or brain network reorganization.

The enhanced FC between the VAN and other networks observed in the right DLPFC-tDCS protocol simulations (Fig 3C) aligns with clinical evidence, demonstrating the tDCS-HBM’s capability to capture network-level neuromodulatory effects across stimulation protocols. VAN is involved in the process of attention reorientation triggered by exogenous stimuli, which is closely related to the individual’s alertness [49–51]. Clinical studies indicated that right DLPFC-tDCS can significantly improve alertness and selective attention [52,53] and the selective attention is associated with an increase in FC of the VAN [54]. These empirical studies therefore provide indirect evidence for our finding that right DLPFC-tDCS stimulation could predict alertness-related FC changes (i.e., FC between VAN network and other networks). This finding also offers valuable insights into electrode placement in clinical tDCS applications. For instance, tDCS targeting the right DLPFC may be a preferred option for patients who require enhanced selective attention and alertness.

Furthermore, the dynamic changes of functional network across three phases revealed by our model also align with previous modeling and empirical studies, demonstrating the validity of tDCS-HBM in predicting tDCS effects and exploring propagation mechanisms. First, in Phase 1 (During-stimulation period),we observed a significant decrease in whole-brain FC, consistent with findings from a previous modeling study using an AD virtual brain network model, which also reported a rapid reduction in functional connectivity at the initial stage of stimulation [27]. They suggested this initial decrease may be related to transient functional connectivity disruption induced by exogenous stimulation [27]. In addition, we found an increase in brain complexity and flexibility during stimulation (Fig 4B). This finding exhibits cross-modal consistency with Wang et al.’s spectral entropy analysis of resting-state EEG, which revealed a sustained increase in spectral entropy from the baseline period (T0) to post-intervention (T3) following 14 consecutive days of HD-tDCS administration [55]. This reflects a transient adjustment process of the brain network as it adapts to the stimulation [27], potentially allowing the brain to flexibly reconfigure its functional networks [56].

In Phase 2 (cover the transition from During-stimulation period to Post-stimulation period), brain regions with strong SC to the anodically stimulated region exhibited significant enhancement in FC first, followed by enhanced whole-brain FC (Figs 4, 5, S6 and S8). We suggest that the initial enhancement of FC in regions exhibiting strong SC with the anodically stimulated region arises from the propagation of electrical stimulation along strong structural pathways (Fig 4A), thereby synchronizing neural activity patterns in these strongly connected regions with the stimulated region. Subsequently, the enhanced whole-brain FC reflects the characteristic of the brain as a complex dynamic system, where inter-regional interactions facilitate coordinated responses across multiple regions [57,58], thereby triggering widespread FC enhancement.

Finally, in Phase 3 (Post-stimulation period), we find that the increased FC gradually disappeared and whole-brain FC recovered to resting-state levels. The transient FC enhancement compared to Without-tDCS condition, which suggest that the complex brain networks can remain highly adaptable and coordination after stimulation ceases [26,59]. Our findings of FC enhancement align with Kunze et al.’s perspective that synchronization is the primary mechanism of tDCS effects [26]. Additionally, Gibson and colleagues indicated that synchronization may profoundly impact GM and WM through changes in synaptic plasticity, axonal caliber and myelination [60]. However, the transient appearance of FC enhancement after stimulation cessation highlights the necessity for long-term tDCS applications to ensure sustained induction and consolidation of neuroplastic changes.

One of the advantages of our study is the consideration of the experimentally observed mechanism that E-fields linearly induce membrane potential polarization. Based on this improvement our model predicts the modulation effect of tDCS over different spatial and temporal scales, which is consistent with previous modeling and clinical studies and enhances our understanding of the propagation mechanisms of tDCS effects. However, there are potential limitations to our current research. First, homogeneous parameter fitting was applied uniformly across all 68 cortical regions, and subcortical structures such as the thalamus and basal ganglia were not taken into account. This approach does not adequately capture the intrinsic dynamic heterogeneity and unique connectivity patterns of cortical and subcortical networks. Therefore, future studies should incorporate spatially heterogeneous parameters and develop coupled cortex–subcortex models to reproduce the complex dynamics of large-scale brain networks. Second, although the modeling results received preliminary support from relevant clinical literature, they should be interpreted with caution. Subsequent work should leverage empirically acquired, concurrently recorded tDCS–fMRI data to optimize the tDCS-HBM parameters, thereby significantly enhancing predictive accuracy and reliability. Finally, tDCS–EEG data should be further integrated to construct a hybrid brain-network model informed by EEG, facilitating a multimodal examination of how electrical stimulation modulates functional brain networks across spatial and temporal scales and providing a stronger theoretical foundation for the design of individualized stimulation protocols.

## Conclusion

In conclusion, we proposed a tDCS-HBM framework that maps the electric field to equivalent membrane potential via a linear relationship and modulates synaptic gating variables based on the polarization level of membrane potential, thereby predicting brain activity in response to tDCS. The observed similarities and differences in brain functional networks under two DLPFC-tDCS protocols indicate that tDCS-HBM is promising for predicting the network effects of various tDCS protocols. The dFC results further reveal that the propagation effects of tDCS are mediated by SC. The consistency of the tDCS effects and their propagation mechanism with existing modeling and empirical studies further validates the validity of tDCS-HBM.

## Materials and methods

### The tDCS hybrid brain model

#### Large-scale brain network model.

We utilized a large-scale brain network model of 68 neural masses to simulate brain dynamics activity. The MFM proposed by Deco et al. [61] was used as a neural mass model to characterize the dynamics of local cortical regions. Coupling between 68 cortical regions is determined by SC. The MFM was derived by applying mean-field reductions to a spiking neural network model, which incorporates firing rates and synaptic gating dynamics [62]. Each cortical region was represented by subnetworks of coupled excitatory and inhibitory populations of spiking neurons, described by the following set of nonlinear stochastic differential equations:

$${\dot{S}}_{i}=-\frac{S_{i}}{\tau_{S}}+\gamma \left(1-S_{i}\right)H\left(x_{i}\right)+\sigma v_{i}\left(t\right),$$ (1)

$$x_{i}=wJS_{i}+GJ\sum_{j}C_{ij}S_{j}+I,$$ (2)

$$H\left(x_{i}\right)=\frac{ax_{i}-b}{1-\exp\left(-d\left(ax_{i}-b\right)\right)},$$ (3)

Where *S*_*i*_, *x*_*i*_, and *H*(*x*_*i*_) denote the average synaptic gating variable, the total input current, and the average firing rate of each population in the cortical region *i*, respectively. The parameters are derived from values extracted from neurophysiological data to ensure the biophysical realism of the model [63]. In the large-scale brain network model developed in this study, we employed a homogeneous parameter-fitting strategy, applying a single set of model parameters to all 68 cortical regions. Detailed parameter definitions and the fitting procedure [33] are provided in the Supporting Information. With the above dynamic MFM, we show that an empirically calibrated, dynamic, and mutually coupled whole-brain model can simulate system-level brain dynamics activity. The detailed fitting results of the simulated and empirical data are provided in the Supporting information (S9 and S10 Figs).

#### tDCS E-fields model.

In this study, we utilized the open-source SimNIBS (v4.0) software [63] to simulate the electric fields (E-fields) induced by tDCS. We focused on studying the effects of tDCS on the DLPFC using two tDCS protocols for active stimulation of the left DLPFC (F3a-Fp2c, EEG10-10, Anodal F3, Cathodal Fp2, electrode size 5×7 cm^2^, 2 mA) and the right DLPFC (F4a-Fp1c, EEG10-10, Anodal F4, Cathodal Fp1, electrode size 5×7 cm^2^, 2 mA). See the Supporting Information for details on the E-field modeling.

We aim to identify brain regions with the strongest electric fields (E-fields) (crucial brain regions) under the two stimulation protocols, which we then used as stimulated brain regions (anodically stimulated regions and cathodally stimulated regions) in a large-scale brain network model. Considering that anodal excitation and cathodal inhibition under scalp electrodes are outdated and simplistic [64], we utilized the E-field strength in the cortex to determine the brain regions most affected by stimulation. The normal and tangential components of the E-fields (S11 Fig) on the cerebral cortex, calculated by SimNIBS [65], were mapped onto a gray matter mesh. We define the 99th percentile as the peak electric field based on the E-fields. Assuming that the maximum $\left|{\vec{E}}_{\text{normal}}\right|$ (the normal components of the E-fields, see Supporting Information) of a given brain region is larger than the peak electric field, this brain region is then considered to be the crucial brain region affected by tDCS (crucial regions, see Supporting Information S1 Table).

#### Incorporating E-fields into the large-scale brain network model.

To account for the effects of tDCS, we relied on the fact that E-fields affect neurons in a geometry-dependent fashion [28]. The field effect is maximized when the direction of the externally applied E-field is parallel to the main axis of the cell (corresponding to ${\vec{E}}_{\text{normal}}$), whereas the field effect is zero when the direction of the E-field is orthogonal to the direction of the cell (corresponding to ${\vec{E}}_{\text{tangent}}$) [24–26]. Furthermore, it has been shown that fields aligned with the direction of the dendritic tuft to axon produce a positive (depolarizing or excitatory) perturbation of the membrane potential at the soma [24–26] (corresponding to the surface inward ${\vec{E}}_{\text{normal}}$). Conversely, a field in the reverse direction produces a negative effect (hyperpolarization or inhibition, corresponding to the surface outward ${\vec{E}}_{\text{normal}}$) [24–26]. Previous studies described this effect of transcranial current stimulation (tCS) on the neuronal population level as the “λE model” [24,26].

In the tDCS-HBM, the above considerations lead us to consider the tDCS effect as a linear function of the external E-fields applied to the average membrane potential of the neuronal population. That is,$V_{\text{tDCS}}=\lambda {\vec{E}}_{\text{normal}}$, where *λ* is the linear coefficient of the externally applied E-field induced membrane polarization, and ${\vec{E}}_{\text{normal}}$ is the normal E-field of the chosen crucial brain region. It has been shown that during anodal and cathodal stimulation of tDCS, the net polarizing effect of tDCS on the type P sub-population was limited to no more than |4mV|, an amount of polarization that corresponds to an E-field polarization of 30 mV/mm in in-vivo experiments [24,26,28]. Therefore, we set *λ* to be 0.13. This bias voltage term $V_{\text{tDCS}}$ may have a depolarizing or hyperpolarizing effect on a given subpopulation. We introduce a modulation factor θ(i) into the synaptic gating dynamics to quantitatively characterize the regulatory effects of tDCS-induced membrane potential polarization on the gating variable opening fraction. When θ(i)>0, membrane potential depolarization enhances the NMDA receptor channel opening proportion; when θ(i)<0, membrane potential hyperpolarization reduces the NMDA receptor channel opening proportion.

$$V_{tDCS}\left(i\right)=\lambda {\vec{E}}_{normal}\left(i\right),$$ (4)

$$\theta (i)=kV_{tDCS}\left(i\right)$$ (5)

$${\dot{S}}_{i}=-\frac{S_{i}}{\tau_{S}}+\gamma (1-S_{i})H(x_{i})+\sigma v_{i}\left(t\right)+\theta (i),$$ (6)

Where *i* refers to a cortical brain region, ${\vec{E}}_{\text{normal}}$ denotes the region-wise averaged normal component of the E-field (parallel to the neuron), obtained by mapping the SimNIBS simulation results onto the Desikan-Killiany atlas (S2 Table). Based on the well-adjusted parameters of large-scale brain network model, which can successfully simulate the system-level brain dynamics. Here, we simplify the relationship between the deviation of membrane potential from resting state and synaptic gating variables into a positive correlation. The value of k was set to 1. During stimulation, the tDCS-modulated regulatory term θ(i) incorporated into synaptic gating variables is maintained as a constant input, which is directly set to zero upon cessation of stimulation.

### BOLD fMRI signal simulation

We utilized structural T1-weighted MRI, dMRI, and resting-state fMRI (rs-fMRI) from 45 subjects in the HCP Retest data for this study. The dataset details and group-level templates are described in the Supporting Information. Using the trained dynamic MFM parameters, we simulated the BOLD signals under the left DLPFC-tDCS, right DLPFC-tDCS, and Without-tDCS conditions, with 30 runs for each condition. The initial 60 seconds of the simulated BOLD signals were removed to eliminate the initial transient. Subsequently, a stimulus lasting 60 seconds was applied. The BOLD time series was downsampled to 0.72 seconds to match the temporal resolution of the empirical BOLD signals from the HCP (a total of 1200 time points). We have included results for different stimulation durations (e.g., 30s, 90s, or 120s, see S12, S13, S14 and S15 Figs) in the Supporting Information, which demonstrate consistency across stimulation durations and indicate a tendency for enhancement to be more pronounced with longer stimulation durations.

### Cortical parcellation

We obtained 68 cortical regions by cortical segmentation based on the brain atlas defined by Desikan et al. Then, the overlapping areas on the surface of the cerebral cortex were evaluated and the 68 cortical regions were divided into 7 resting state networks (RSNs) based on the 7 RSNs defined by Yeo et al [66]. The seven networks were named as follows: frontoparietal control network (FPN), default mode network (DMN), limbic network (LIM), dorsal attention network (DAN), ventral attention network (VAN), sensorimotor network (SMN), and visual network (VIS). This mapping enables a more systematic and precise evaluation of the effects and spatial distribution of stimulation or model simulation results across different functional brain networks.

To validate the reproducibility of our findings, we further employed the Destrieux atlas [67] to construct personalized dynamic models and perform virtual stimulation analyses. The Destrieux atlas segments the cerebral cortex into 148 regions, which were assigned to one of the seven resting-state networks (RSNs) defined by Yeo et al [66] based on the extent of cortical surface overlap.

### Structural connectivity

We constructed each subject’s structural connectivity (SC) matrix using probabilistic tractography. Specifically, diffusion MRI data from 45 participants were first preprocessed according to the HCP minimal preprocessing pipeline [68]; fiber tracking was then performed using the MRtrix3 toolkit [69]. Each subject yielded a weighted 68×68 SC matrix, where the weight corresponded to the number of tracks between two regions normalized by the total cortical area. For the group-averaged SC matrix, to mitigate false-positive connections arising from subject-specific tractography noise, we retained only those connections present in at least 50% of participants and averaged their weights across subjects [70]. Finally, to ensure parameter stability and comparability, the resulting group SC matrix was scaled so that its maximum element is 0.2.

### DLPFC-tDCS effects on brain network

To investigate the effects of the stimulation from two DLPFC-tDCS protocols on brain functional networks, we conducted a comprehensive analysis of brain activity following tDCS from a static perspective. We selected the BOLD signal of the first 130 TRs as the analyzed data (S16 Fig) to avoid annihilation of the tDCS effect. First, we calculated differences in whole-brain FC (see Supporting Information) between the two DLPFC-tDCS protocols and the Without-tDCS condition using a paired sample t-test. Then, we calculated three indicators from a global perspective: global efficiency (as a measure of network integration), clustering coefficient (as a measure of network segregation), and the average shortest path length (overall efficiency of information integration in the brain). Next, we measured the effects of tDCS at the subnetwork level by calculating the strength of FC within (cohesion of functional networks) and between RSNs (inter-network integration capacity of functional networks). Finally, we investigated how the strength of SC between the whole-brain and the anodically stimulated region influenced their FC at the nodal level. The brain regions were categorized into “High-SC” (top 10 regions with the stronger SC to anodically stimulated region) and “Low-SC” (remaining 57 regions). We compared the t-values of FC changes between the anodically stimulated region and brain regions with either High-SC or Low-SC following two DLPFC-tDCS protocols. We only considered brain regions showing significant differences in FC.

To further explore how the strength of SC between whole-brain and anodically stimulated regions affects changes in BOLD signal upon stimulation, we calculated the correlation between the changes in BOLD (across three periods) and the strengths of SC between whole-brain and the anodically stimulated region. Specifically, we first segmented the BOLD signals into three periods: Without-tDCS, During-stimulation, and Post-stimulation (S16 Fig). Then, we calculated the significant differences between the mean BOLD values of each brain region (excluding the anodically and cathodically stimulated regions) across three periods (During vs. Without, During vs. Post, and Without vs. Post) using a paired sample t-test. Finally, we used Spearman correlation analysis to explore the association between the t-value of the change in BOLD signal in whole-brain (excluding the anodically and cathodically stimulated regions) across three periods and their SC (the 20% threshold) to the anodically stimulated region (p≤0.05). Results obtained with alternative sparsity thresholds (30%–50%) are presented in Supplementary S17 Fig and reveal similarly robust, positive SC–BOLD correlations, demonstrating the consistency of our findings across thresholding schemes. The absence of significant BOLD differences in Post > Without contrasts precluded further analysis of their associations with SC. Moreover, to investigate the effect of stimulation on the brain’s capacity for information processing, we calculated the Hurst exponent, Lempel-Ziv complexity, permutation fuzzy entropy (PFEN), metastability, and synchrony indicators to observe the complexity and flexibility of the brain network across three periods (Without-tDCS, During-stimulation, and Post-stimulation).

To elucidate the propagation mechanism of the tDCS effects, we computed whole-brain dFC based on simulated BOLD signal time series using a sliding-window technique (S16 Fig). We used a time window of 40 TRs (time points) with a step of 1 TR to compute dFC. As a result, a weighted 3D adjacency matrix (68 × 68 × 1161) was obtained, where 68 denotes the number of cortical brain regions and 1161 denotes the number of sliding windows. The dFC results at different values of the time window (e.g., 30TRs and 50TRs, S18 and S19 Figs) are available in the Supporting Information. In order to better understand the transition process of the brain’s spatiotemporal dynamics from the During-stimulation to the Post-stimulation period, we divided the sliding window into three phases. Phase 1: the window slides throughout the During-stimulation period. Phase 2: the window includes the transition from the During-stimulation to the Post-stimulation period. Phase 3: the window slides throughout the Post-stimulation period. Finally, to further elucidate the influence of SC on the propagation of the tDCS effects, we investigated how the strength of SC between whole-brain and the anodically stimulated region affects the t-value of their FC change.

### Statistical analysis

All statistical analyses were performed in SPSS (IBM SPSS Statistics 25) in this study. Paired samples t-tests were used to assess significant differences in global, subnetwork, nodal, and dFC metrics between the Without-tDCS (30 runs) and the two DLPFC-tDCS (30 runs) protocols, followed by False Discovery Rate (FDR) correction for multiple comparisons. For indicators of brain complexity and flexibility, paired samples t-tests were used to assess significant differences across the Without-tDCS, During-stimulation, and Post-stimulation scenarios, with FDR correction applied for multiple comparisons. The significance threshold was set at p≤0.05.

## Supporting information

S1 Fig Stimulation-induced changes in whole-Brain functional connectivity based on the Destrieux Atlas. (EPS)

S2 Fig Stimulation-induced changes in functional connectivity within and between brain networks based on the Destrieux Atlas. (EPS)

S3 Fig Brain complexity and flexibility in right DLPFC-tDCS protocol (F4a-Fp1). Data are expressed as *Mean* ± *SD*. Statistical comparisons were made between conditions (without-tDCS, during-stimulation, and after stimulation) using a paired-samples *t*-test. *: *p* < 0.05, **: *p* < 0.01, ***: *p* < 0.001.
(EPS)

S4 Fig Changes in BOLD Signal, Flexibility, and Complexity across three periods of left DLPFC-tDCS (F3a-Fp2c), based on the Destrieux Atlas. (EPS)

S5 Fig Brain synchronization over time in left DLPFC-tDCS protocol (F3a-Fp2c). The horizontal axis marks the time window, and the vertical axis represents the t-value obtained from a paired samples t-test of synchrony at different periods vs. without-tDCS.
(EPS)

S6 Fig Dynamic network effects of right DLPFC-tDCS protocol (F4a-Fp1c). The sliding windows are divided into three phases. Phase 1: The window slides throughout the During-stimulation period. Phase 2: the window covers the transition from the During-stimulation to the Post-stimulation period. Phase 3: the window slides throughout the Post-stimulation period. In each phase, five time-varying FC matrices (depicted as small rectangles in the figure) are shown, where each FC matrix represents the statistical comparison using a paired sample t-tests between the left DLPFC-tDCS protocol and the Without-tDCS condition. Only the FC in the left DLPFC-tDCS protocol that is significantly different from Without-tDCS (paired sample t-test) is colored, while the remaining are white. Warmer colors indicate an increase in FC, while cooler colors indicate a decrease in FC. Color bars indicate statistical t-values (*p* < 0.05).
(EPS)

S7 Fig Dynamic functional connectivity changes in the left DLPFC-tDCS protocol (F3a-Fp2c) based on the Destrieux Atlas. (EPS)

S8 Fig Impact of structural connectivity on dynamic functional connectivity in stimulated regions in right DLPFC-tDCS protocol (F4a-Fp1c). A Impact of structural connectivity on dynamic functional connectivity in anodically stimulated region. B Impact of structural connectivity on dynamic functional connectivity in cathodically stimulated region. Horizontal coordinates represent sliding windows over time, while vertical coordinates represent cortical regions with weak-to-strong (bottom-to-top) structural connections to the anodal or cathodal stimulated cortical brain regions. Only the FC in the tDCS protocol that is significantly different from without tDCS (paired samples t-test) is colored, while the remaining nodes are white. Warmer colors correspond to an increase in functional connectivity, while cooler colors correspond to a decrease in functional connectivity. The color bars indicate statistical t-values (*p* < 0.05).
(EPS)

S9 FigThe fitting between simulated and empirical data in large-scale brain network models.This study first conducted model inversion on both individual and group-level models across 45 participants. A demonstrates the correlation between simulated FC and empirical FC for individual models (r=0.487±0.076, p < 0.0001), where model-empirical FC consistency significantly exceeded the empirical SC-FC correlation (r=0.239±0.049, p < 0.0001) across all participants. B demonstrated that the goodness-of-fit between model simulations and empirical data is significantly higher than that of randomly generated null model distributions. C presents the group-level model performance, showing strong correlation between simulated FC and empirical group-level FC (r = 0.72, p < 0.0001). D illustrates progressive ICC improvement with repeated simulations: mean pairwise ICC reached 0.4257 for single simulations, increasing to $ICC_{ave\_5}=0.7209$, $ICC_{ave\_10}=0.8531$, and $ICC_{ave\_30}=0.9381$ with 5, 10, and 30 simulation averages, respectively.(EPS)

S10 Fig Spatial patterns of functional connectivity in simulated and empirical data. It illustrates the comparative organization patterns of intra-RSN and inter-RSN connectivity strengths between simulated and empirical data. Both group-level and individual models successfully captured the prototypical spatial configurations observed in empirical datasets, demonstrating robust alignment with neurobiological ground-truth patterns.
(EPS)

S11 Fig Visualization of tDCS current modeling. A indicates the placement of the F4a-Fp1c electrode montages, respectively, with red indicating the anode and blue the cathode. B represents the magnitude of the E-field in the cerebral cortex. C denotes the normal E-field in the cerebral cortex. All panels were rendered using the SimNIBS software package [30].
(EPS)

S12 Fig Network effects of DLPFC-tDCS protocols with different stimulation duration. The left corresponds to the F3a-Fp2c DLPFC-tDCS protocol, and the right corresponds to the F4a-Fp1c DLPFC-tDCS protocol. Statistical comparisons were made between the tDCS protocol and without-tDCS using a paired-samples t-test (*p* < 0.05). Only nodes with significant differences (paired-samples t-test) are colored, while the remaining nodes are white. Warmer colors indicate an increase in FC strength after the stimulation, while cooler colors indicate a decrease in FC strength following the stimulation. The color bar indicates the statistical t-value.
(EPS)

S13 Fig Dynamic network effects of left DLPFC-tDCS protocol (F3a-Fp2c) with the stimulation duration of 30s (42 TRs). The sliding windows are divided into three phases. Phase 1: The window slides throughout the During-stimulation period. Phase 2: the window covers the transition from the During-stimulation to the Post-stimulation period. Phase 3: the window slides throughout the Post-stimulation period. In each phase, five time-varying FC matrices (depicted as small rectangles in the figure) are shown, where each FC matrix represents the statistical comparison using a paired sample t-tests between the left DLPFC-tDCS protocol and the Without-tDCS condition. Only the FC in the left DLPFC-tDCS protocol that is significantly different from Without-tDCS (paired sample t-test) is colored, while the remaining are white. Warmer colors indicate an increase in FC, while cooler colors indicate a decrease in FC. Color bars indicate statistical t-values (*p* < 0.05).
(EPS)

S14 Fig Dynamic network effects of left DLPFC-tDCS protocol (F3a-Fp2c) with the stimulation duration of 90s (125 TRs). The sliding windows are divided into three phases. Phase 1: The window slides throughout the During-stimulation period. Phase 2: the window covers the transition from the During-stimulation to the Post-stimulation period. Phase 3: the window slides throughout the Post-stimulation period. In each phase, five time-varying FC matrices (depicted as small rectangles in the figure) are shown, where each FC matrix represents the statistical comparison using a paired sample t-tests between the left DLPFC-tDCS protocol and the Without-tDCS condition. Only the FC in the left DLPFC-tDCS protocol that is significantly different from Without-tDCS (paired sample t-test) is colored, while the remaining are white. Warmer colors indicate an increase in FC, while cooler colors indicate a decrease in FC. Color bars indicate statistical t-values (*p* < 0.05).
(EPS)

S15 Fig Dynamic network effects of left DLPFC-tDCS protocol (F3a-Fp2c) with the stimulation duration of 120s (168 TRs). The sliding windows are divided into three phases. Phase 1: The window slides throughout the During-stimulation period. Phase 2: the window covers the transition from the During-stimulation to the Post-stimulation period. Phase 3: the window slides throughout the Post-stimulation period. In each phase, five time-varying FC matrices (depicted as small rectangles in the figure) are shown, where each FC matrix represents the statistical comparison using a paired sample t-tests between the left DLPFC-tDCS protocol and the Without-tDCS condition. Only the FC in the left DLPFC-tDCS protocol that is significantly different from Without-tDCS (paired sample t-test) is colored, while the remaining are white. Warmer colors indicate an increase in FC, while cooler colors indicate a decrease in FC. Color bars indicate statistical t-values (*p* < 0.05).
(EPS)

S16 Fig Measurement method schematic. A represents the BOLD data used to analyze the tDCS effects on brain functional network. B represents the BOLD data used to analyze the whole-brain BOLD activities and brain complexity. C represents the BOLD data used to analyze the propagation of DLPFC-tDCS effects in dFC.
(EPS)

S17 Fig Correlation between changes in BOLD signal and structural connectivity at 20%—50% sparsity thresholds. (EPS)

S18 Fig Dynamic network effects of left DLPFC-tDCS protocol (F3a-Fp2c) with a sliding window length of 30 TRs. The sliding windows are divided into three phases. Phase 1: The window slides throughout the During-stimulation period. Phase 2: the window covers the transition from the During-stimulation to the Post-stimulation period. Phase 3: the window slides throughout the Post-stimulation period. In each phase, five time-varying FC matrices (depicted as small rectangles in the figure) are shown, where each FC matrix represents the statistical comparison using a paired sample t-tests between the left DLPFC-tDCS protocol and the Without-tDCS condition. Only the FC in the left DLPFC-tDCS protocol that is significantly different from Without-tDCS (paired sample t-test) is colored, while the remaining are white. Warmer colors indicate an increase in FC, while cooler colors indicate a decrease in FC. Color bars indicate statistical t-values (*p* < 0.05).
(EPS)

S19 Fig Dynamic network effects of left DLPFC-tDCS protocol (F3a-Fp2c) with a sliding window length of 50 TRs. The sliding windows are divided into three phases. Phase 1: The window slides throughout the During-stimulation period. Phase 2: the window covers the transition from the During-stimulation to the Post-stimulation period. Phase 3: the window slides throughout the Post-stimulation period. In each phase, five time-varying FC matrices (depicted as small rectangles in the figure) are shown, where each FC matrix represents the statistical comparison using a paired sample t-tests between the left DLPFC-tDCS protocol and the Without-tDCS condition. Only the FC in the left DLPFC-tDCS protocol that is significantly different from Without-tDCS (paired sample t-test) is colored, while the remaining are white. Warmer colors indicate an increase in FC, while cooler colors indicate a decrease in FC. Color bars indicate statistical t-values (*p* < 0.05).
(EPS)

S1 Table Crucial Regions. (PDF)

S2 Table Electric Field (F3a-Fp2c) Mapping Results in Desikan-Killiany Atlas Regions. (PDF)

S1 FileSupporting Information.This document elucidates the methodological frameworks encompassing: (1) Large-scale brain network model detail, (2) tDCS E-field detail, (3) Caleulation of ${\vec{E}}_{\text{normal}}$, (4) multimodal neuroimaging dataset detail, (5) Functional connectivity (FC), and (6) Outcome measures detail-metastable.(PDF)
