# Supplementary material for: Effects of tDCS of the DLPFC on brain networks: A hybrid brain modeling study
Source: PLoS Comput Biol. 2025 Sep 16;21(9):e1013486. doi: 10.1371/journal.pcbi.1013486 (PMC12456829; doi:10.1371/journal.pcbi.1013486)
Supplement: S1 Table — (PDF) [file pcbi.1013486.s020.pdf]

S1 Table Crucial Regions

protocols	99% peak electric filed	Crucial areas ($\vec{E}_{normal}$)	Anodal stimulation regions ($avg(\vec{E}_{normal})$)	Cathodal stimulation regions ($avg(\vec{E}_{normal})$)
F3a-Fp2c (left DLPFC-tDCS)	0.340 V/m	LOF R (-0.3408 V/m); rMFG L (0.3766 V/m)	rMFG L (0.0525 V/m)	LOF R (-0.0561 V/m);
F4a-Fp1c (right DLPFC-tDCS)	0.347 V/m	LOF L (-0.3470 V/m); rMFG R (0.3472 V/m)	rMFG R (0.0609 V/m)	LOF L (-0.0484 V/m);
