# Supplementary material for: Effects of tDCS of the DLPFC on brain networks: A hybrid brain modeling study
Source: PLoS Comput Biol. 2025 Sep 16;21(9):e1013486. doi: 10.1371/journal.pcbi.1013486 (PMC12456829; doi:10.1371/journal.pcbi.1013486)
Supplement: S2 Table — (PDF) [file pcbi.1013486.s021.pdf]

S2 Table Electric Field Mapping Results in Desikan-Killiany Atlas Regions

ID	Area_mm2	StructName	Mean (V/m)	Min (V/m)	Max (V/m)
1	1204.8	bankssts	0.0096	-0.1137	0.1302
2	639.7	caudalanteriorcingulate	0.005	-0.1294	0.124
3	2737.3	caudalmiddlefrontal	0.0236	-0.2023	0.2963
4	1335.2	cuneus	-0.006	-0.0729	0.0674
5	518.3	entorhinal	-0.0544	-0.138	0.0821
6	3384.4	fusiform	-0.018	-0.1474	0.113
7	4584.8	inferiorparietal	0.0097	-0.0959	0.116
8	4235.5	inferiortemporal	-0.0133	-0.1303	0.1251
9	1121.2	isthmuscingulate	-0.0291	-0.1582	0.0794
10	5611.2	lateraloccipital	0.005	-0.0869	0.0899
11	2865.2	lateralorbitofrontal	-0.0182	-0.2339	0.2246
12	2832.4	lingual	-0.0217	-0.1142	0.0719
13	2097.3	medialorbitofrontal	-0.0645	-0.2571	0.1188
14	3654.7	middletemporal	0.0205	-0.1251	0.1186
15	775.9	parahippocampal	-0.0348	-0.1121	0.0606
16	1762.5	paracentral	-0.0683	-0.1522	0.0737
17	1928.9	parsopercularis	0.0371	-0.2565	0.288
18	709.6	parsorbitalis	-0.0286	-0.2362	0.2346
19	1662.8	parstriangularis	0.0338	-0.1955	0.2489
20	1462.2	pericalcarine	-0.0141	-0.0954	0.0742
21	5149.3	postcentral	0.0074	-0.1991	0.2518
22	1241.5	posteriorcingulate	0.0127	-0.1336	0.1155
23	5635.7	precentral	0.0294	-0.2749	0.2582
24	4383.1	precuneus	-0.0239	-0.1201	0.0787
25	774.1	rostralanteriorcingulate	-0.0981	-0.196	0.0684
26	6696.1	rostralmiddlefrontal	0.0525	-0.2981	0.3766
27	7909.2	superiorfrontal	-0.0287	-0.2919	0.3297
28	5802.6	superiorparietal	0.0192	-0.1006	0.1505
29	4479.8	superiortemporal	0.0274	-0.1212	0.1819
30	4651.9	supramarginal	0.0141	-0.17	0.1863
31	202.3	frontalpole	-0.0616	-0.2158	0.1363
32	462.9	temporalpole	-0.0289	-0.1096	0.0838
33	526.6	transversetemporal	0.0174	-0.0738	0.1226
34	2516.6	insula	0.0838	-0.1833	0.1874
35	1004.6	bankssts	-0.0079	-0.0783	0.0649
36	822	caudalanteriorcingulate	0.0822	-0.0905	0.1845
37	2446.5	caudalmiddlefrontal	0.0129	-0.1913	0.1666
38	1524	cuneus	0.0175	-0.0617	0.0729
39	453.4	entorhinal	0.0354	-0.0963	0.1569
40	3672.1	fusiform	0.0013	-0.1153	0.1713
41	5768.6	inferiorparietal	-0.0007	-0.0807	0.0887
42	3603.7	inferiortemporal	-0.0102	-0.1577	0.1231
43	1058.3	isthmuscingulate	0.0221	-0.0839	0.1387
44	6007.9	lateraloccipital	-0.0062	-0.0654	0.0639
45	2814.1	lateralorbitofrontal	-0.0561	-0.3408	0.3353

46	3247	lingual	-0.0015	-0.1042	0.1109
47	2113.2	medialorbitofrontal	0.0631	-0.2386	0.3028
48	3894.6	middletemporal	-0.0049	-0.1287	0.1293
49	705.3	parahippocampal	0.0044	-0.0632	0.0637
50	1970.7	paracentral	0.0315	-0.0432	0.1395
51	1529.2	parsopercularis	-0.0079	-0.1902	0.1311
52	867.6	parsorbitalis	-0.0711	-0.2483	0.1602
53	1741.9	parstriangularis	-0.0124	-0.2539	0.3026
54	1430.6	pericalcarine	0.0049	-0.0681	0.0924
55	4384.2	postcentral	-0.0016	-0.1523	0.109
56	1342.8	posteriorcingulate	0.0504	-0.1201	0.1651
57	5638	precentral	-0.0042	-0.144	0.1606
58	4570.1	precuneus	0.0197	-0.0679	0.111
59	674.2	rostralanteriorcingulate	0.0528	-0.0948	0.1594
60	6414.7	rostralmiddlefrontal	-0.0606	-0.3289	0.2842
61	7747.9	superiorfrontal	0.0352	-0.3137	0.3119
62	6429.1	superiorparietal	-0.0019	-0.0748	0.0918
63	4156.1	superiortemporal	-0.0097	-0.1284	0.1245
64	3536.1	supramarginal	-0.004	-0.1516	0.1021
65	243	frontalpole	0.0346	-0.2074	0.2673
66	479.3	temporalpole	-0.0246	-0.2376	0.1375
67	394.6	transversetemporal	-0.0044	-0.0611	0.0432
68	2745.9	insula	-0.0743	-0.2065	0.0729
