# Supplementary material for: Effects of tDCS of the DLPFC on brain networks: A hybrid brain modeling study
Source: PLoS Comput Biol. 2025 Sep 16;21(9):e1013486. doi: 10.1371/journal.pcbi.1013486 (PMC12456829; doi:10.1371/journal.pcbi.1013486)
Supplement: S1 File — This document elucidates the methodological frameworks encompassing: (1) Large-scale brain network model detail, (2) tDCS E-field detail, (3) Caleulation of E→normal, (4) multimodal neuroimaging dataset detail, (5) Functional connectivity (FC), and (6) Outcome measures detail-metastable. (PDF) [file pcbi.1013486.s022.pdf]

Supporting Information

(1) Large-scale brain network model detail

S_i, x_i and $H(x_i)$ denote the average synaptic gating variable, the total input current and the average firing rate of each population in cortical area i, respectively. The neuronal population firing rate $H(x_i)$ is determined by the total input current x_i, and the rest are excitatory gating variables, where $a = 270(VnC)$, $b = 108(Hz), d = 0.154(s)$. The kinetic parameters are $\gamma =$ 0.641/1000 (the factor 1000 is for expressing everything in ms), and τ_S=100 ms. The $v_i(t)$ is uncorrelated standard Gaussian noise and the noise amplitude at each node is σ. The total input current x_i consists of three components. The first component, wJS_i, represents the retrograde excitatory transmission generated by the activity of the neuronal population S_i, where $J = 0.2609(nA)$ for the the strength of the reciprocal coupling of superimposed excitatory and inhibitory synapses in the local microloop, i.e., the effective conductance of the local excitatory-inhibitory neural circuit, scaled by the coupling factor w, and S_i is the local neuronal population synaptic gating variable. The second component $GJ\sum_j C_{ij}S_j$ represents the overall excitatory input to other areas coupled to cortical area i, where G is the anatomical link based on the SC matrix, which represents the relationship between area i and the region j white matter connection density, with the strength of the coupling of the structural connections scaled by the global coupling factor G. The third component, I, represents the total subcortical input of excitability. These parameters are taken from Deco et al. from the original peak neural network model proposed by Brunel and Wang[1], which uses values from neurophysiological data to achieve biophysical realism.

The dynamic mean-field model captures the average neurodynamic behavior of cortical regions at the level of neuronal populations using interpretable dynamic variables and physiological parameters such as population firing rates and average synaptic gating variables. The simulated neural activities of each cortical region Si were transformed to a BOLD signal[2] using the Balloon-Windkessel hemodynamic model[3]. Note though that this is a simple approximation to more detailed hemodynamic models. Each brain region i is governed by the following nonlinear differential equations:

$$\dot{z}_i = S_i - \kappa z_i - \gamma(f_i - 1) \tag{1}$$

$$\dot{f}_i = z_i \tag{2}$$

$$\dot{v}_i = \frac{1}{\tau}\left(f_i - v_i^{1/\alpha}\right) \tag{3}$$

$$\dot{q}_i = \frac{1}{\tau}\left\{\frac{f_i}{\rho}\left[1 - (1-\rho)^{1/f_i}\right] - q_i v_i^{\frac{1}{\alpha}-1}\right\} \tag{4}$$

$$\dot{Y}_i = V_0\left[k_1(1-q_i) + k_2\left(1 - \frac{q_i}{v_i}\right) + k_3(1 - v_i)\right] \tag{5}$$

where zi, fi, vi, qi, and Yi represent the vasodilatory signal, blood inflow, blood volume, deoxyhemoglobin content, and BOLD-fMRI signal, respectively. The model parameters and their values are defined as follows: signal decay rate κ = 0.65 s^{-1}, elimination rate γ = 0.41 s^{-1}, hemodynamic transit time τ = 0.98 s, Grubb's exponent α = 0.32, resting oxygen extraction fraction ρ = 0.34, resting blood volume fraction V0 = 0.02, and fMRI parameters k1 = 3.72, k2 =

0.53, and k3 = 0.53. The parameter values were taken from 3T using Appendix A of Heinzle et al[4].

The MFM and Balloon-Windkessel hemodynamic model were simulated using the euler method with an integration step size of 10 ms. We ran the simulation for 16.4 min with the neural activities Si initialized randomly. We discarded the first 2 min of simulation time to remove initial transients and downsampled the time series to 0.72 s to have the same temporal resolution as the empirical BOLD signals from the HCP.

To estimate the optimal set of model parameters—including the synaptic coupling strength www and external input III for each cortical region, as well as the global coupling parameter G and noise intensity σ—we employed a Bayesian inversion framework. The fitting procedure aimed to maximize the Pearson correlation coefficient between the upper triangular elements of the simulated functional connectivity (FC) matrix and the empirical FC derived from resting-state fMRI data. Specifically, we adopted the Expectation-Maximization (EM) algorithm[5] to iteratively optimize the model parameters and enhance the correspondence between simulated and empirical FC. Based on prior knowledge and previous studies, the initial values of the parameters were set as follows: w=0.5, I=0.3 G=1, and σ=0.001. Additional methodological details can be found in the Supplementary Information and in the study by Wang et al[6].

In the large-scale brain network model developed in this study, we employed a homogeneous parameter-fitting strategy, applying a single set of model parameters to all 68 cortical regions. With the above dynamic MFM, we show that an empirically calibrated, dynamic, and mutually coupled whole-brain model can simulate system-level brain dynamics activity. The detailed fitting results of the simulated and empirical data are provided in the Supporting Information (S5 Fig, S6 Fig).

(2) tDCS E-field detail

It has been verified in the literature that SimNIBS with other electric field modeling software (i.e., ROAST) and intracranial recordings with similar accuracy of electric fields between different modeling approaches[7]. Since we used a large-scale brain network model based on the HCP group-level data, in order to eliminate the impact of individualized data, we chose to use the dataset provided by SimNIBS for finite element modeling. Specifically, the T1w and T2w structural MRI included in the SimNIBS example dataset was used to perform tissue segmentation with reconstruction of the tetrahedral head mesh through the charm pipeline, and the contours were visually checked for accuracy through the final tissue segmentation viewer. Then, virtual electrodes were placed on the scalp based on the EEG10-10 system. Conductivity values were then assigned to each tissue (white matter: 0.126 S/m, gray matter: 0.275 S/m, cerebrospinal fluid: 1.654 S/m, bone: 0.010 S/m, scalp: 0.465, spongy bone: 0.010 S/m, compact bone: 0.008 S/m, and sponge: 0.008 S/m, compact bone: 0.008 S/m, eye balls: 0.500 S/m, eye region: 0.250 S/m, electrode rubber: 29 S/m, electrode saline: 1.5 S/m)[8, 9, 10].The thicknesses of the electrodes and sponges were modeled with 1.5 mm and 2 mm, respectively. The electric field was calculated using the SimNIBS software finite element method and visualized using gmsh[11].

To align the cortical electric field (E-field) distributions generated by SimNIBS with a standard brain atlas, we employed a two-step processing pipeline based on surface resampling and atlas-based parcellation.

First, the surface-based normal component of the E-field ($\vec{E}_{normal}$) was resampled from the individual head model space to the standard fsaverage template using the mri_surf2surf utility

provided by the FreeSurfer software package. This resampling relied on the sphere.reg file to establish anatomically consistent vertex-wise correspondence across surfaces, thereby preserving the anatomical integrity of the cortical E-field distribution during transformation.

Next, to assign E-field values to anatomical regions, we used the mri_segstats tool to parcellate the fsaverage cortical surface according to the Desikan–Killiany (DK) atlas (e.g., aparc.a2009s). Within each atlas-defined region, we computed two metrics: (1) the maximum $\vec{E}_{normal}$ across all included vertices to evaluate peak stimulation effects; and (2) the average $\vec{E}_{normal}$ across all vertices in the region to reflect overall stimulation strength.

To identify the most significantly stimulated regions, we further calculated the 99th percentile of $\vec{E}_{normal}$ across all cortical vertices. Any region containing at least one vertex exceeding this threshold was labeled as a "significantly stimulated region." The average E-field of these regions was then used as region-specific external input to the dynamic mean field (DMF) model, providing a physiologically informed basis for simulating stimulation-induced network dynamics.

(3) Calculation of $\vec{E}_{normal}$

At first, the normal electric field surface vertices calculated by SimNIBS were aligned with the surface vertices of Freesurfer segmentation. Then, the normal electric field surface was further processed in the Freesurfer toolbox by dividing it into 68 brain regions based on the Desikan-Killiany brain atlas and calculating the average normal electric field.

(4) Multimodal neuroimaging dataset detail

A database managed by the Washington University Minnesota (WU-Minn) consortium of the Human Connectome Project (HCP: http://www.humanconnectome.org). All imaging data was acquired on a specially customized 3T Siemens connectome-Skyra scanner with a 32-channel head coil. Each participant completed two rs-fMRI scans (voxel size: 2.0 × 2.0×2.0 mm^3, FoV: 208 × 180 mm^2, 72 slices, TR = 720 ms, TE = 33.1 ms, flip angle = 52°) over a two-day period, with each scan consisting of two resting-state runs, each lasting approximately 14 minutes (1200 time points). The T1-weighted structural image was acquired using a three-dimensional magnetization-prepared rapid acquisition gradient echo image (MPRAGE) sequence (voxel size: 0.7 × 0.7×0.7 mm^3, FoV: 224 × 224mm^2, 320 slices, TR = 2400 ms, TE = 2.14 ms, flip angle = 8°). The T2 anatomical images were recorded in the same resolution (voxel size: 0.7 × 0.7×0.7 mm^3, FoV: 224 ×224 mm^2, 320 slices) and with a TR of 3200 ms and a TE of 565ms. The dMRI data were acquired by diffusion weighting of the three shells using b-values = 1000, 2000 and 3000 s/mm^2. Details of the data collection can be found on the HCP website http://www.humanconnectome.org/).

(5) Functional connectivity

The rs-fMRI time series were preprocessed based on the HCp minimal preprocessing function pipeline processing[12]. Next, the rs-fMRI time series were segmented into 68 regions by Desikan-Killiany brain atlas. Finally, we calculated the correlation of regional blood oxygen level dependent (BOLD) time series using Pearson correlation coefficients to obtain functional connectivity.

(6) Outcome measures detail-metastable

We calculated three network topology metrics, global efficiency, clustering coefficient, and

characteristic path length, based on the GRETNA software package. Information exchange requires synchronization between brain network regions. Synchronization is a common method for studying the brain's information transfer capacity, and metastable dynamics balance the competing demands of information transfer in the system. These behaviors maximize information flow and capacity. The first step in quantifying the phase synchronization of two or more time series is to determine their instantaneous phases, and the most commonly used method is the Hilbert transform, which does a good job of establishing the intrinsic connection between the original signal and the phase and amplitude components obtained after the Hilbert transform. Furthermore, compared with other phase synchronization analysis methods (e.g., time-domain analysis, frequency-domain analysis, and wavelet analysis), the Hilbert transform is more reflective of the realism of the data, and is more in line with the characteristics of biological signals. To calculate phase synchronization using the Hilbert transform method, the signal is first band-pass filtered around a frequency of interest, and then the Hilbert transform is applied to obtain the instantaneous phase. For the bandpass filtered BOLD signal $f(t)$, it can be interpreted in terms of an analytic function expression. Assume that this analytic signal is $F(t)$, which is defined as shown in Equation (1):

$$F(t) = f(t) + iH[f(t)] \quad (1)$$

where $i = \sqrt{-1}$, $H[(t)]$ is the value of the real signal after the Hilbert transform. The principle of Hilbert transform is as follows:

$$H[f(t)] = \frac{1}{\pi}\int_{-\infty}^{+\infty}\frac{f(\tau)}{t-\tau}d\tau \quad (2)$$

In another way, the analytic signal $F(t)$ can also be expressed by Equation (3).

$$F(t) = A(t)e^{i\theta(t)} \quad (3)$$

where $\theta(t)$ and $A(t)$ represent the instantaneous phase and instantaneous amplitude of the real signal, respectively. The instantaneous phase value of this signal is obtained by calculating the arctangent of the ratio of the imaginary time series to the real time series, which takes the range $[-\pi, \pi]$. Equation (4) shows the following.

$$\theta(t) = arctan\frac{H[f(t)]}{f(t)} \quad (4)$$

The instantaneous phase information of the BOLD signal was extracted after the Hilbert transform, yielding the phase evolution of the BOLD time course for each brain region. To obtain the whole-brain pattern of BOLD phase coherence at each single time point t, a dynamic phase-locking matrix $dPC(n, p, t)$ was computed, which estimates the phase alignment between nodes n and p of each pair of brain regions at each time t, as shown in Equation (5).

$$dPC(n, p, t) = \cos(\theta(n, t) - \theta(p, t)) \quad (5)$$

The "instantaneous" collective behavior of the global phase oscillator can be described by the Kuramoto order parameter $R(t)$, which is calculated as:

$$R(t) = \frac{1}{N}\left|\sum_{k=1}^{N} e^{i\theta_k(t)}\right| \quad (6)$$

where $k = 1,2,3..., N$ represents the number of brain regions, and $\theta_k(t)$ is the instantaneous phase of the oscillator k at time t. In the case of complete independence, all distributions are uniform and $R(t)$ is close to 0. In contrast, if all phases are equal, $R(t)$ is close to 1. **Here, global metastable is defined as the standard deviation of the time series $R(t)$ for all brain regions, and the global synchrony is mean of $R(t)$.**

TABLE1 Resting-state network assignments of Desikan-Killiany atlas

RSN	Number	Brain regions name	Acronyms
DMN	1	Bankssts L	L.BSTS
	2	Inferiorparietal L	L.IPL
	3	Isthmuscingulate L	L.iCC
	4	Middletemporal L	L.MTG
	5	Parahippocampal L	L.paraH
	6	Parsorbitalis L	L.pORB
	7	Posteriorcingulate L	L.PCC
	8	Precuneus L	L.PCUN
	9	Rostralanteriorcingulate L	L.ACC
	10	Superiorfrontal L	L.SF
	11	Bankssts R	R.BSTS
	12	Inferiorparietal R	R.IPL
	13	Isthmuscingulate R	R.iCC
	14	Middletemporal R	R.MTG
	15	Parahippocampal R	R.paraH
	16	Parsorbitalis R	R.pORB
	17	Posteriorcingulate R	R.PCC
	18	Precuneus R	R.PCUN
	19	Rostralanteriorcingulate R	R.rACC
	20	Superiorfrontal R	R.SF
FPN	21	Caudalmiddlefrontal L	L.cMFG
	22	Rostralmiddlefrontal L	L.rMFG
	23	Caudalmiddlefrontal R	R.cMFG
	24	Rostralmiddlefrontal R	R.rMFG
LIM	25	Entorhinal L	L.ENT
	26	Inferiortemporal L	L.ITG
	27	Lateralorbitofrontal L	L.LOF
	28	Medialorbitofrontal L	L.MOF
	29	Frontalpole L	L.PF
	30	Temporalpole L	L.TP
	31	Entorhinal R	R.ENT
	32	Inferiortemporal R	R.ITG
	33	Lateralorbitofrontal R	R.LOF
	34	Medialorbitofrontal R	R.MOF
	35	Frontalpole R	R.PF
	36	Temporalpole R	R.TP
VAN	37	Caudalanteriorcingulate L	L.cACC
	38	Parsopercularis L	L.pOPER
	39	Parstriangularis L	L.pTRI
	40	Supramarginal L	L.SMAR
	41	Insula L	L.INS
	42	Caudalanteriorcingulate R	R.cACC
	43	Parsopercularis R	R.pOPER
	44	Parstriangularis R	R.pTRI
	45	Supramarginal R	R.SMAR
	46	Insula R	R.INS
DAN	47	Superiorparietal L	L.SPL
	48	Superiorparietal R	R.SPL
SMN	49	Paracentral L	L.paraC
	50	Postcentral L	L.postC
	51	Precentral L	L.preC
	52	Superiortemporal L	L.STG
	53	Transversetemporal L	L.TT
	54	Paracentral R	R.paraC
	55	Postcentral R	R.postC
	56	Precentral R	R.preC
	57	Superiortemporal R	R.STG
	58	Transversetemporal R	R.TT
VIS	59	Cuneus L	L.CUN
	60	Fusiform L	L.FUS
	61	Lateraloccipital L	L.LOG

	62	Lingual L	L.LING
	63	Pericalcarine L	L.periCAL
	64	Cuneus R	R.CUN
	65	Fusiform R	R.FUS
	66	Lateraloccipital R	R.LOG
	67	Lingual R	R.LING
	68	Pericalcarine R	R.periCAL

References

[1]. Brunel N, Wang X-J. Effects of Neuromodulation in a Cortical Network Model of Object Working Memory Dominated by Recurrent Inhibition. *Journal of Computational Neuroscience* **11**, 63-85 (2001). https://doi.org/10.1023/A:1011204814320

[2]. Gordon EM, *et al.* Precision functional mapping of individual human brains. **95**, 791-807. e797 (2017).

[3]. Friston KJ, Harrison L, Penny WJN. Dynamic causal modelling. **19**, 1273-1302 (2003).

[4]. Heinzle J, Koopmans PJ, den Ouden HE, Raman S, Stephan KEJN. A hemodynamic model for layered BOLD signals. **125**, 556-570 (2016).

[5]. Friston KJJN. Bayesian estimation of dynamical systems: an application to fMRI. **16**, 513-530 (2002).

[6]. Wang P, *et al.* Inversion of a large-scale circuit model reveals a cortical hierarchy in the dynamic resting human brain. **5**, eaat7854 (2019).

[7]. Sahib AY, Seyedarabi H, Afrouzian R, Farhoudi M. A MATLAB-Based Toolbox to Simulate Transcranial Direct-Current Stimulation Using Flexible, Fast, and High Quality Tetrahedral Mesh Generation. *IEEE Access* **10**, 76573-76585 (2022). https://doi.org/10.1109/access.2022.3190410

[8]. Saturnino GB, Antunes A, Thielscher A. On the importance of electrode parameters for shaping electric field patterns generated by tDCS. *NeuroImage* **120**, 25-35 (2015). https://doi.org/10.1016/j.neuroimage.2015.06.067

[9]. Antonenko D, *et al.* Towards precise brain stimulation: Is electric field simulation related to neuromodulation? *Brain Stimulation* **12**, 1159-1168 (2019). https://doi.org/10.1016/j.brs.2019.03.072

[10]. Thielscher A, Opitz A, Windhoff M. Impact of the gyral geometry on the electric field induced by transcranial magnetic stimulation. *NeuroImage* **54**, 234-243 (2011). https://doi.org/10.1016/j.neuroimage.2010.07.061

[11]. Müller D, Habel U, Brodkin ES, Clemens B, Weidler C. HD-tDCS induced changes in resting-state functional connectivity: Insights from EF modeling. *Brain Stimulation* **16**, 1722-1732 (2023). https://doi.org/10.1016/j.brs.2023.11.012

[12]. Glasser MF, *et al.* The minimal preprocessing pipelines for the

Human Connectome Project. *NeuroImage* **80**, 105-124 (2013). https://doi.org/10.1016/j.neuroimage.2013.04.127
